# Thyroid transcription factor-1 as a prognostic indicator for stage IV lung adenocarcinoma with and without EGFR-sensitizing mutations

**DOI:** 10.1186/s12885-019-5792-0

**Published:** 2019-06-13

**Authors:** Ji Young Park, Seung Hun Jang, Hwan Il Kim, Joo-Hee Kim, Sunghoon Park, Yong Il Hwang, Ki-Suck Jung, Jinwon Seo, Chang Youl Lee, Yousang Ko, Yong-Bum Park

**Affiliations:** 1 Department of Pulmonary, Allergy and Critical Care Medicine, Hallym University Sacred Heart Hospital, Anyang, Republic of Korea; 2 Lung Research Institute of Hallym University College of Medicine, Chuncheon, South Korea; 3 Pathology, Hallym University Sacred Heart Hospital, Anyang, Republic of Korea; 4 Department of Pulmonary, Allergy and Critical Care Medicine, Chuncheon Sacred Heart Hospital, Hallym University, Chuncheon, Republic of Korea; 5 Department of Pulmonary, Allergy and Critical Care Medicine, Kangdong Sacred Heart Hospital, Hallym University, Seoul, Republic of Korea

**Keywords:** TTF-1 expression, Stage IV lung adenocarcinoma, EGFR-sensitizing mutations

## Abstract

**Background:**

Thyroid transcription factor (TTF)-1 expression is a diagnostic marker and a good prognostic indicator for lung adenocarcinoma. However, its good prognostic ability might be due to epidermal growth factor receptor (EGFR)-sensitizing mutations as shown by the positive correlation between TTF-1 expression and EGFR mutations. We explored the prognostic impact of TTF-1 expression according to EGFR-sensitizing mutation status in lung adenocarcinoma patients.

**Methods:**

We conducted a retrospective cohort study of patients with stage IV lung adenocarcinoma. Data were extracted from the lung cancer registry of Hallym University Medical Centers (three hospitals) in Korea between March 2006 and March 2016.

**Results:**

Overall, 173 patients were included. EGFR-sensitizing mutations were detected in 84 (51.4%) patients. TTF-1 expression was positive in 139 (80.3%) patients; it was significantly correlated with EGFR-sensitizing mutations (*p* < 0.001). TTF-1-positive lung adenocarcinoma patients had longer overall survival (OS) than those who were TTF-1 negative (19.3 vs. 5.8 months, *p* < 0.001). In a Cox regression analysis, TTF-1 positivity, Stage IV M1a, good performance status, and EGFR-sensitizing mutations were independently associated with prolonged OS. In the subgroup of wild-type EGFR adenocarcinoma patients, TTF-1 positivity was also a good prognostic indicator for OS and progression-free survival (PFS) after first-line cytotoxic chemotherapy.

**Conclusions:**

TTF-1 expression was a good prognostic indicator for OS and PFS in stage IV lung adenocarcinoma patients with and without EGFR-sensitizing mutations.

**Electronic supplementary material:**

The online version of this article (10.1186/s12885-019-5792-0) contains supplementary material, which is available to authorized users.

## Background

Despite several advances in cancer diagnosis and treatment, the prognosis of advanced lung cancer remains poor. In previous studies, patient-related factors such as performance, age, and female sex were identified as independent prognostic indicators of lung cancer stage [1]. Although there are many studies on prognostic biomarkers, most of them did not adjust for previous prognostic factors. Similar to epidermal growth factor receptor (EGFR) mutations, some specific driving mutations for target treatment are not only predictive factors for outcomes but also play a prognostic role [2]. Targeted therapy is a standard treatment modality in lung adenocarcinoma even in elderly patients or those with a poor performance status [3]. As such, more objective independent prognostic biomarkers are needed in clinical practice and in studies on lung cancer; this is particularly true for lung adenocarcinoma because of its increasing incidence worldwide [4].

Thyroid transcription factor (TTF)-1 is a tissue-specific transcription factor that has a homeodomain protein fold and regulates the expression of select genes in the thyroid and lung for embryonic development and differentiation [5]. The importance of TTF-1 extends into adulthood as it plays a critical role in maintaining the normal function of terminal respiratory unit cells by controlling surfactant proteins [6]. TTF-1 is a lineage marker and has been used as a diagnostic marker for lung adenocarcinoma and small cell carcinoma [7]. A subsequent study showed that TTF-1 overexpression was a favorable prognostic marker among patients with lung adenocarcinoma [8]. Anagnostou et al. showed that TTF-1 expression positively impacted the survival of stage I lung adenocarcinoma patients [9]. Although several studies also demonstrated similar results in advanced stage lung adenocarcinoma, there were some limitations such as small sample sizes or an uncontrolled driving mutation status. Chung et al. showed that TTF-1 was an independent prognostic factor among patients treated with EGFR tyrosine kinase inhibitors (TKI). However, they did not control for treatment lines, and EGFR-TKI treatment was not based on the EGFR mutation status [10]. The prognostic significance of TTF-1 among patients with wild-type EGFR adenocarcinoma has not been studied adequately. Moreover, research on the prognosis of patients with TTF-1–negative, EGFR-positive adenocarcinoma is also limited. The good prognostic capability of TTF-1 expression might be due to EGFR-sensitizing mutations as demonstrated by the positive correlation between TTF-1 expression and EGFR mutations [10, 11]. The purpose of this study was to explore the prognostic impact of TTF-1 expression based on the EGFR-sensitizing mutation status in lung adenocarcinoma patients.

## Methods

### Study population and design

We retrospectively examined a cohort of consecutive patients with lung adenocarcinoma from the registry of Hallym Medical Centers (three teaching hospitals) in Korea, between March 2006 and March 2016. Patients were included in this study if they 1) had stage IV (7th edition of the TNM Classification) lung adenocarcinoma at the time of initial diagnosis with both TTF-1 immunohistochemistry (IHC) and EGFR mutation results available, 2) had an Eastern Cooperative Oncology Group (ECOG) performance status score of 0–2, and 3) were receiving systemic anti-cancer treatment (cytotoxic chemotherapy or targeted treatment). All included patient tissues were subjected to IHC staining to detect TTF-1 expression, and clamping polymerase chain reaction (PCR) was used to detect EGFR gene mutations. Patients who had a poor performance status, discontinued anti-cancer treatment (chemotherapy or target treatment) due to non-medical problems, had more than one type of primary cancer, received surgery as primary treatment, were pathologically diagnosed with adenosquamous cell carcinoma, or had ALK fluorescence in situ hybridization positivity were excluded. Clinical and genetic data, pathological data, and follow-up information were retrieved from the cohort database. The Response Evaluation Criteria in Solid Tumors were used to assess tumor response and disease progression [12]. Progression-free survival (PFS) was defined as the period from the first day of cancer treatment to the date of disease progression or the date of death due to any cause. Overall survival (OS) was calculated as the time from the first day of cancer treatment to death due to any cause or to the last follow-up on July 20, 2017.

### TTF-1 IHC and EGFR mutation detection

The tissue specimens were obtained using different diagnostic modalities such as percutaneous needle biopsies, bronchoscopic biopsies, transbronchial needle aspirations, and surgery. TTF-1 expression was assessed as part of the routine diagnostic evaluation. The formalin-fixed paraffin-embedded (FFPE) tumor tissue blocks were divided into 4-μm-thick sections used for IHC. After dewaxing and rehydration, the tissue slide was incubated with monoclonal anti-TTF-1 (8G7G3/1, Dako, Carpinteria, CA, USA; Dilution 1:100). If any positive nuclear staining (not only cytoplasmic staining) was identified, it was recorded as TTF-1 positive staining. Genomic DNA was extracted from the FFPE tissue blocks using the High Pure PCR Template Preparation Kit (Roche Diagnostics). The PNAClamp™ EGFR Mutation Detection Kit (Panagene, Daejeon, Korea), which uses peptide nucleic acid mediated real-time PCR clamping technology, was used to detect EGFR mutations [13]. Amplification was performed using the CFX96 real-time system (BioRad, Hercules, CA, USA).

### Data analysis

Statistical analyses of categorical variables were performed using chi-squared or Fisher’s exact tests. Continuous variables were analyzed using Student’s t-tests or Mann–Whitney U-tests. The Kaplan-Meier method was used to estimate overall survival, progression-free survival. A Cox proportional hazards model was used to estimate the effect of TTF-1 expression on survival, adjusting for baseline characteristics. A *p*-value < 0.05 was considered statistically significant. All analyses were performed using SPSS for Windows ver. 18.0 (SPSS, Chicago, IL, USA).

## Results

### Baseline characteristics

Of the 697 patients with lung adenocarcinoma, 224 patients were in stage IV and had results on both TTF-1 IHC and EGFR mutations. Five patients with ALK rearrangement were excluded. An additional 46 patients were excluded due to poor performance status or the discontinuation of anti-cancer treatment as a result of non-medical problems. Therefore, in total 173 patients were included in the analysis. The clinical features of the study patients are summarized in Table 1. The mean age was 65.9 ± 11.7 years, and 68 (39.3%) were women. Ninety-eight (56.6%) patients were current or former smokers. All patients had histologically confirmed stage IV (M1a: 30.1%; M1b: 69.9%) adenocarcinoma. EGFR-sensitizing mutations were detected in 84 (51.4%) patients. TTF-1 expression was positive in 139 (79.8%) patients. TTF-1 positivity was more frequent in patients who were female, had good performance scores, had never smoked, and had an EGFR-sensitizing mutation (Table 1). Of these factors, TTF-1 expression was the most strongly correlated with an EGFR-sensitizing mutation (*p* < 0.001).

**Table 1:** Clinical characteristics of the study population (*n* = 173)

	TTF-1 positive (*n* = 139)	TTF-1 negative (*n* = 34)	Total (n = 173)	*P* value
Age (mean ± SD), years	65.8 ± 11.4	66.4 ± 12.8	65.9 ± 11.7	0.787
< 70	75 (81.5)	17 (18.5)	92 (53.2)	0.679
= or > 70	64 (79.0)	17 (21.0)	81 (46.8)	
Female, no. (%)	62 (91.2)	6 (8.8)	68 (39.3)	0.004
Smoking status, no. (%)				0.001
Never smoker	69 (92.0)	6 (8.0)	75 (43.4)	
Current or former smoker	70 (71.4)	28 (28.6)	98 (56.6)	
Stage, no. (%)				0.745
M1a	41 (78.8)	11 (21.2)	52 (30.1)	
M1b	98 (81.0)	23 (19.0)	121 (69.9)	
ECOG status, no. (%)				0.009
0	67 (89.3)	8 (10.7)	75 (43.4)	
1–2	72 (73.5)	26 (26.5)	98 (56.6)	
EGFR sensitizing mutation, no. (%)				< 0.001
Wild-type	60 (67.4)	29 (32.6)	89 (51.4)	
Mutation	79 (94.0)	5 (6.0)	84 (48.6)	

*Abbreviations*: *SD* standard deviation, *TTF-1* thyroid transcription factor 1, *ECOG* Eastern Cooperative Oncology Group, *EGFR* epidermal growth factor receptor

### EGFR mutation patterns

The EGFR mutation patterns are shown in Table 2. Among the 84 patients with EGFR mutations, 44 (52.4%) and 33 (39.3%) had exon 19 and 21 (L858R) deletions, respectively. One patient initially had triple EGFR mutations that included a deletion in exon 19, G719X, and de novo T790 M. There were no patients with duplication or insertion in exon 20. Twenty-seven patients underwent re-biopsies for disease progression after EGFR-TKI treatments. Among them, 15 (55.6%) patients had acquired T790 M mutations. All biopsy specimens from these patients with acquired T790 M mutations were TTF-1 positive.

**Table 2:** Mutation patterns in 84 patients with mutant EGFR stratified based on TTF-1 status

EGFR mutations	TTF-1 positive (*n* = 79) n (%)	TTF-1 negative (*n* = 5) n (%)	Total (*n* = 84) n (%)	*P* value
Exon 18 G719X	8 (10.1)	1 (20.0)	9 (10.7)	0.441
Exon 19 deletion	43 (54.4)	1 (20.0)	44 (52.4)	0.187
Exon 21 L858R	30 (38.0)	3 (60.0)	33 (39.3)	0.377
de novo T790 M	1 (1.3)	0 (0)	1 (1.2) ^a^	1.000
Acquired T790 M	15/27 (55.6)	0 (0)	15/27 (55.6)	NA

*Abbreviations*: *EGFR* epidermal growth factor receptor, *TTF-1* thyroid transcription factor 1

^a^One patient initially had triple EGFR mutations, which included deletion in exon 19, G719X, and de novo T790 M

### TTF-1 expression and OS

**Fig. 1:**
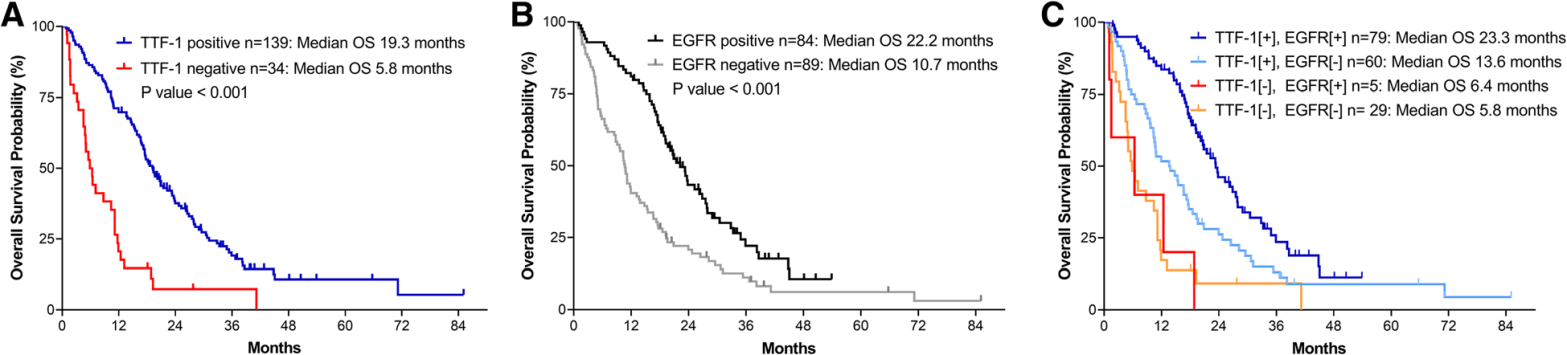
Survival stratified by TTF-1 expression (**a**), EGFR mutation status (**b**), and by both TTF-1 and EGFR mutations (**c**). Abbreviations: TTF-1, thyroid transcription factor-1; EGFR, epidermal growth factor receptor

Patients with TTF-1-positive lung adenocarcinoma had longer OS than those with TTF-1-negative malignancy (19.3 vs. 5.8 months, *p* < 0.001) (Fig. 1). Table 3 shows the Cox proportional hazards model for OS. The crude hazard ratio (HR) of the TTF-1 positive group compared to the negative group was 0.395 (95% [confidence interval] CI, 0.254–0.615, *p* < 0.001). Additionally, the ECOG score (*p* = 0.001), EGFR-sensitizing mutations (*p* = 0.022), and Stage IV M1a (*p* = 0.005) were significantly associated with survival. Five patients had EGFR mutation-positive and TTF-1-negative adenocarcinoma. The mean PFS for EGFR-TKI was short (2.9 months) among these patients and OS was also significantly lower than in the patients positive for TTF-1 and EGFR mutations (6.4 vs. 23.3 months, *p* < 0.001).

**Table 3:** Cox proportional hazards model of overall survival for the 173 patients with lung adenocarcinoma

Predictor variable vs. reference variable	Univariate analysis		Multivariate analysis	
	*P* value	HR (95% CI)	*P* value	Adjusted HR (95% CI)
Age (< 70) vs. ≥ 70	0.147	0.783 (0.562–1.090)	0.557	0.897 (0.625–1.288)
Female vs. male	0.001	0.562 (0.398–0.793)	0.410	0.763 (0.400–1.454)
Never smoker vs. ever smoker	0.001	0.563 (0.400–0.792)	0.794	0.917 (0.478–1.759)
Stage IV, M1a vs. M1b	0.103	0.738 (0.513–1.063)	0.005	0.586 (0.405–0.849)
ECOG 0 vs. 1–2	< 0.001	0.461 (0.325–0.654)	0.001	0.522 (0.356–0.764)
EGFR sensitizing mutation vs. wild type	< 0.001	0.497 (0.355–0.696)	0.022	0.640 (0.438–0.937)
TTF-1 positive vs. negative	< 0.001	0.319 (0.212–0.480)	< 0.001	0.395 (0.254–0.615)

*Abbreviations*: *HR* Hazard ratio, *CI* confidence intervals, *ECOG* Eastern Cooperative Oncology Group, *EGFR* epidermal growth factor receptor, *TTF-1* thyroid transcription factor 1

### Cytotoxic chemotherapy response

**Fig. 2:**
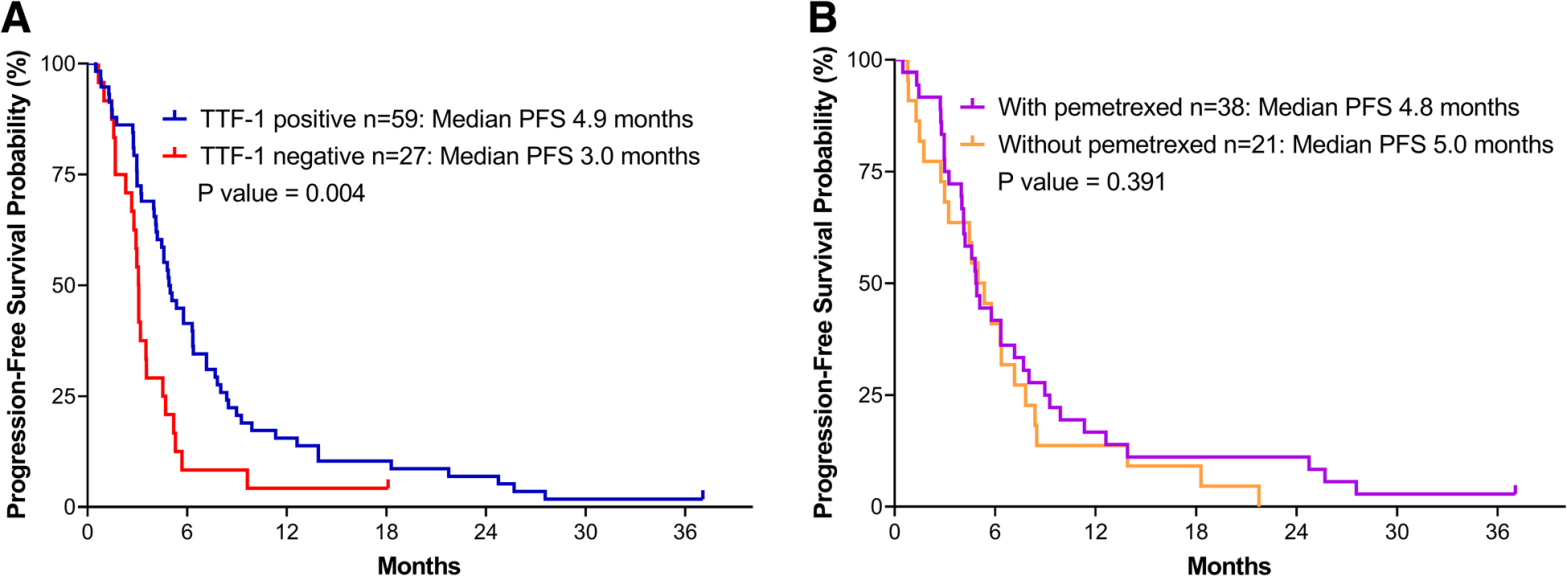
PFS in patients who received cytotoxic chemotherapy as first-line treatment stratified by TTF-1 status (**a**) and in patients with TTF-1-positive expression who received cytotoxic chemotherapy as first-line treatment with or without pemetrexed (**b**). Abbreviations: TTF-1, thyroid transcription factor-1; PFS, progression free survival

The tumor response for cytotoxic chemotherapy as the first-line treatment could be evaluated in 86 patients (TTF-1 positive, 59 cases; TTF-1 negative, 27 cases; EGFR mutation, 2 cases) (Fig. 2). No patient achieved complete remission (CR), while 37, 34, and 15 achieved partial remission (PR), stable diseases (SD), and progressive disease (PD), respectively. The objective response rate (CR + PR) and disease control rate (CR + PR + SD) did not significantly differ between the TTF-1-positive and negative groups (Table 4). However, the PFS for initial cytotoxic treatment was longer in patients with TTF-1-positive lung cancer than in those with TTF-1-negative lung cancer (PFS: 4.9 months vs. 3.0 months, *p* = 0.004). Among the 59 patients with TTF-1-positive malignancy, 38 received pemetrexed-based treatments, while 21 received chemotherapy without pemetrexed (Additional file 1: Table S1). The disease control rate was higher in the pemetrexed group than that in the non-pemetrexed group (97.7% vs. 71.4%, *p* = 0.019). However, there were no differences in OS and PFS between the two groups (*p* > 0.05). Among the 27 patients with TTF-1-negative lung cancer, there were no significant differences in the OS, PFS, and chemotherapy response rates between the patients treated with and without pemetrexed (*p* > 0.05, Additional file 1: Table S2).

**Table 4:** Response analysis among patients who received cytotoxic chemotherapy as first-line treatment stratified by TTF-1 status

	TTF-1 positive (*n* = 59)	TTF-1 negative (*n* = 27)	Total (*n* = 86)	*P* value
EGFR sensitizing mutation (%)	2 (3.4)	0 (0)	2 (2.3)	1.000
Response, no. (%)				
Objective response rate (CR + PR)	27 (45.8)	10 (37.0)	37 (43.0)	0.448
Disease control rate (CR + PR + SD)	51 (86.4)	20 (74.1)	71 (82.6)	0.161
PFS (months), median (95% CI)	4.9 (3.9–5.8)	3.0 (2.8–3.2)	4.4 (3.7–5.2)	0.004

*Abbreviations*: *EGFR* epidermal growth factor receptor, *CR* complete remission, *PR* partial remission, *SD* stable diseases, *PFS* progression free survival, *CI* confidence interval

### TTF-1 expression among patients with EGFR-wild type lung adenocarcinoma

In the subgroup of patients with wild-type EGFR adenocarcinoma, the median OS of patients with TTF-1 positive expression was significantly longer compared to those with TTF-1 negative expression (13.6 months vs. 5.8 months, *p* = 0.005). Multivariate analysis showed that TTF-1 positivity was the strongest prognostic factor for OS (HR 0.51; 95% CI: 0.31–0.83; *p* = 0.006), and for PFS among patients who received first-line cytotoxic chemotherapy (HR 0.49; 95% CI, 0.29–0.81; *p* = 0.006) (Additional file 1: Table S3).

## Discussion

In this study, we demonstrated that TTF-1 expression was a good prognostic indicator for OS and PFS in patients with stage IV lung adenocarcinoma regardless of the presence or absence of EGFR mutations. We also confirmed that TTF-1 positivity was strongly correlated with EGFR mutations. However, it is also of note that EGFR mutation positivity and TTF-1 expression negativity did not guarantee a good response of EGFR-TKI.

TTF-1 is a homeodomain nuclear transcription protein of the NKX2 gene family. By binding to specific gene sequences, TTF-1 modulates the transcriptional activation of target genes [5]. TTF-1 is expressed in type II pneumocytes and Clara cells and it regulates the surfactant and Clara cell secretory protein gene expression to maintain normal lung functions [14]. However, the role of TTF-1 in lung cancer pathogenesis and biology is uncertain. Some data suggest that TTF-1 might promote carcinogenesis by enhancing cell proliferation, namely at least adenocarcinoma [15–17]. The NKX2–1 locus, which encodes TTF-1, is frequently amplified in the lung cancer genome [18]. TTF-1 could be important for the survival of a subset of patients with lung adenocarcinomas expressing TTF-1 based on the lineage-specific dependency model [19]. TTF-1 knockdown via RNA interference in these adenocarcinoma cell lines substantially induced tumor growth inhibition and apoptosis [17, 19]. In contrast, the results of both previous studies and the present study indicate that TTF-1 was associated with prolonged survival in patients with lung adenocarcinoma. There is quite a bit of evidence suggesting that TTF-1 plays paradoxical tumor-suppressive roles. Myosin-binding protein H (MYBPH) is one of the transcriptional targets of TTF-1. MYBPH inhibits actomyosin organization, which in turn reduces single cell motility and increases collective cell migration. MYBPH activation eventually results in inhibition of cancer invasion and metastasis [20]. Winslow et al. showed that TTF-1 downregulation in adenocarcinoma was related to a loss of differentiation and increased metastatic potential [21]. In their mouse model, NKX2–1-negativity was pathognomonic of high-grade, poorly differentiated tumors. NKX2–1 knockdown allowed the formation of more liver nodules after intrasplenic injection and more lung nodules after intravenous transplantation. In lung adenocarcinoma, TTF-1 plays not only an oncogenic role, but also a suppressive role for progression to an invasive condition while maintaining a minimum degree of differentiation paradoxically.

EGFR sensitive mutations have been associated with similar clinical characteristics to TTF-1 positive expression in patients with lung adenocarcinoma, particularly among women and non-smokers. Our data showed a significant correlation between TTF-1 and EGFR mutations, which is consistent with previous findings. Sheffield et al. demonstrated that TTF-1 expression and EGFR mutations were strongly correlated (*p* < 0.001) [22]. Shanzhi et al. also showed that TTF-1 expression in patients with lung adenocarcinoma was correlated with EGFR mutations [11]. They also demonstrated that the EGFR exon 21 mutation was more significantly correlated with TTF-1 overexpression than the exon 19 mutation. However, our data did not show a significant difference in the correlation of TTF-1 overexpression with specific mutations. From the molecular aspect, Yamaguchi et al. showed that TTF-1 transcriptional activation enhanced and sustained the pro-survival EGFR downstream pathway by inducing receptor tyrosine kinases such as orphan receptor 1 (ROR1) and activating c-Src [23]. They also noted that ROR1 knockdown induced growth inhibition not only of the EGFR sensitive mutation cell line, but also of the first-generation EGFR-TKI resistance T790 M mutation cell line. Recently, Clarke et al. showed that EGFR knockdown led to NKX2–1 upregulation, suggesting a negative feedback loop [16]. In our study, all patients with the acquired T790 M mutation after EGFR-TKI treatment were TTF-1 positive. This could be interpreted that the secondary acquired driving EGFR mutant still needs the TTF-1 pathway signal. Collectively, these findings suggest that beyond the simple clinical correlation, TTF-1 might play an important role in EGFR-driven lung adenocarcinoma oncogenesis and it might be a biomarker of EGFR oncogenic addiction among patients with EGFR mutation-positive lung adenocarcinoma.

TTF-1 expression might be important among patients with an EGFR-sensitizing mutation. In our study, five patients had EGFR mutation-positive and TTF-1-negative adenocarcinoma (Additional file 1: Table S4). Interestingly, these patients were not responsive to EGFR-TKI and their survival prognosis was significantly reduced. They showed rapid progression upon EGFR-TKI treatment. Loss of the anti-metastatic effect of TTF-1 might explain these poor outcomes. Another explanation is that the EGFR mutation may not be an oncogenic driver but a bystander mutation or there might be intra-tumoral heterogeneity in the EGFR mutation status. TTF-1 positivity could be a surrogate for EGFR mutations in driving oncogenicity in lung cancer patients. This might explain why TTF-1 positivity has a more significant prognostic impact than the EGFR mutation status among patients with advanced lung adenocarcinoma in the EGFR-TKI era [24]. Compared with previously documented factors such as good performance status, EGFR mutations, age, sex, smoking status, and distant metastasis, TTF-1 positivity has the most significant prognostic impact in advanced lung adenocarcinoma.

Pemetrexed combination treatment showed better efficacy in patients with non-squamous cell carcinoma than squamous cell carcinoma [25]. However, the mechanisms for such phenomenon are not well documented, and there is no validated predictive marker for pemetrexed treatment outcomes other than the histologic type. One retrospective study demonstrated that TTF-1 expression was a good marker for higher response rates and prolonged PFS and OS in patients with non-squamous, non-small-cell lung cancer [26]. However, these results should be interpreted with caution because they included patients who underwent first-line EGFR-TKI treatment and only examined EGFR mutations in approximately 50% of the studied patients. Moreover, the chemotherapy regimens were not controlled among the patients. The first-line pemetrexed-based chemotherapy was administered to only 21% of patients. Our results did not indicate that TTF-1 was a predictive marker for treatment outcomes following first-line pemetrexed-based therapy among patients with EGFR-negative adenocarcinoma. Recently, Schilsky et al. also showed TTF-1 expression was not predictive of the clinical benefit from pemetrexed-based treatment in patients with adenocarcinoma [24]. Further well-designed prospective studies to evaluate whether TTF-1 is a good response marker for specific chemotherapy regimens should be conducted.

## Conclusions

Our present study indicated that TTF-1 expression was a good prognostic indicator for OS and PFS in patients with stage IV lung adenocarcinoma regardless of the presence or absence of EGFR mutations. Moreover, even in EGFR mutation-positive cases, the EGFR mutation might not be the driving oncogene if TTF-1 expression is negative. EGFR-TKI should be used with caution in these patients. These results should be validated further in well-designed prospective studies, particularly molecular studies.

## Additional file

Additional file 1: **Table S1.** Response analysis among 59 patients with TTF-1 positive expression who received cytotoxic chemotherapy as first-line treatment with or without pemetrexed. **Table S2.** Response analysis among 27 patients with TTF-1 negative expression who received cytotoxic chemotherapy as first-line treatment with or without pemetrexed. **Table S3.** Cox proportional hazard model of overall survival for 89 patients with lung adenocarcinoma harboring wild-type EGFR. **Table S4.** Case summary of five patients with lung adenocarcinoma harboring mutant-type EGFR and no TTF-1 expression. (DOCX 25 kb)

## Data Availability

The dataset used and analysed during the present study is available from the corresponding author upon reasonable request.

## Abbreviations

CI: Confidence interval
CR: Complete remission
ECOG: Eastern Cooperative Oncology Group
EGFR: Epidermal growth factor receptor
OS: Overall survival
PFS: Progression free survival
PR: Partial remission
SD: Stable diseases
TTF-1: Thyroid transcription factor 1
