# Supplementary material for: Thyroid transcription factor-1 as a prognostic indicator for stage IV lung adenocarcinoma with and without EGFR-sensitizing mutations
Source: BMC Cancer. 2019 Jun 13;19:574. doi: 10.1186/s12885-019-5792-0 (PMC6567596; doi:10.1186/s12885-019-5792-0)
Supplement: Supplementary file 1 — Table S1. Response analysis among 59 patients with TTF-1 positive expression who received cytotoxic chemotherapy as first-line treatment with or without pemetrexed. Table S2. Response analysis among 27 patients with TTF-1 negative expression who received cytotoxic chemotherapy as first-line treatment with or without pemetrexed. Table S3. Cox proportional hazard model of overall survival for 89 patients with lung adenocarcinoma harboring wild-type EGFR. Table S4. Case summary of five patients with lung adenocarcinoma harboring mutant-type EGFR and no TTF-1 expression. (DOCX 25 kb) [file 12885_2019_5792_MOESM1_ESM.docx]

Table S1. Response analysis among 59 patients with TTF-1 positive expression who received cytotoxic chemotherapy as first-line treatment with or without pemetrexed

|  | With pemetrexed (n=38) | Without pemetrexed (n=21) | Total  (n=59) | *P* value |
| --- | --- | --- | --- | --- |
| OS, months, median (95% CI) | 14.2 (6.9-21.5) | 14.8 (4.3-25.3) | 14.8 (8.6-21.0) | 0.425 |
| Response, no. (%) |  |  |  |  |
| Objective response rate (CR+PR) | 17 (44.7) | 10 (47.6) | 27 (45.8) | 0.832 |
| Disease control rate (CR+PR+SD) | 36 (94.7) | 15 (71.4) | 51 (86.4) | 0.019 |
| PFS, months, median (95% CI) | 4.8 (4.1-5.6) | 5.0 (3.5-6.5) | 4.9 (3.9-5.8) | 0.391 |

Abbreviations: OS, overall survival; CI, confidence interval; CR, complete remission; PR, partial remission; SD, stable diseases; PFS, progression free survival.

Table S2. Response analysis among 27 patients with TTF-1 negative expression who received cytotoxic chemotherapy as first-line treatment with or without pemetrexed

|  | With pemetrexed (n=15) | Without pemetrexed (n=12) | Total  (n=27) | *P* value |
| --- | --- | --- | --- | --- |
| OS, months, median (95% CI) | 5.8 (4.7-6.8) | 9.7 (4.0-15.4) | 6.8 (4.2-9.4) | 0.737 |
| Response, no. (%) |  |  |  |  |
| Objective response rate (CR+PR) | 4 (26.7) | 6 (50.0) | 10 (37.0) | 0.257 |
| Disease control rate (CR+PR+SD) | 10 (66.7) | 10 (83.3) | 20 (74.1) | 0.408 |
| PFS (months), median (95% CI) | 2.8 (1.6-4.0) | 3.1 (2.8-3.4) | 3.0 (2.8-3.2) | 0.785 |

Abbreviations: OS, overall survival; CI, confidence interval; CR, complete remission; PR, partial remission; SD, stable diseases; PFS, progression free survival.

Table S3. Cox proportional hazard model of overall survival for 89 patients with lung adenocarcinoma harboring wild-type EGFR

| Predictor variable  vs. reference variable | Univariate analysis | | Multivariate analysis | |
| --- | --- | --- | --- | --- |
|  | *P* value | HR (95% CI) | *P* value | HR (95% CI) |
| Age (< 70) vs. ≥ 70 | 0.367 | 0.816 (0.525-1.269) | 0.663 | 0.899 (0.558-1.450) |
| Female vs. male | 0.017 | 0.520 (0.304-0.890) | 0.557 | 0.681 (0.189-2.456) |
| Never smoker vs. ever smoker | 0.015 | 0.532 (0.321-0.884) | 0.877 | 0.910 (0.275-3.009) |
| Stage IV, M1a vs. M1b | 0.033 | 0.602 (0.378-0.961) | 0.015 | 0.558 (0.349-0.894) |
| ECOG 0 vs. 1-2 | 0.014 | 0.557 (0.349-0.889) | 0.094 | 0.638 (0.378-1.079) |
| Pemetrexed, 1^st^ line vs. non-pemetrexed containing regimen | 0.830 | 1.051 (0.669-1.650) | 0.830 | 0.951 (0.599-1.509) |
| TTF-1 positive vs. negative | 0.006 | 0.516 (0.321-0.829) | 0.006 | 0.506 (0.311-0.826) |

Abbreviations: HR, Hazard ratio; CI, confidence intervals, ECOG, Eastern Cooperative Oncology Group; TTF-1, thyroid transcription factor 1.

| Case | Age, year | Sex | Smoking  (pack-years) | ECOG | TNM staging | TTF-1 | EGFR mutation | Treatment (#cycle or #months) | Overall-survival |
| --- | --- | --- | --- | --- | --- | --- | --- | --- | --- |
| #1 | 37 | Male | Smoker (16) | 1 | T4N0M1a | - | L858R | Erlotinib #4 -  Cisplatin (#4) + Pemetrexed #23 | 18.8 months |
| #2 | 60 | Male | Smoker  (40) | 1 | T4N3M1b | - | 19 deletion | Gemcitabine + Cisplatin #4 -  Gefitinib #2 | 6.4 months |
| #3 | 74 | Male | Ex-smoker  (40) | 1 | T4N3M1b | - | G719X | Gefitinib #1 | 1.5 months |
| #4 | 84 | Female | Never smoker | 1 | T4N3M1b | - | L858R | Gefitinib #7 -  Pemetrexed #2 | 12.4 months |
| #5 | 84 | Male | Ex-smoker  (60) | 2 | T3N3M1b | - | L858R | Erlotinib #2 | 1.1 months |

Table S4. Case summary of five patients with lung adenocarcinoma harboring mutant-type EGFR and no TTF-1 expression

TTF-1, thyroid transcription factor 1; ECOG, Eastern Cooperative Oncology Group; EGFR, epidermal growth factor receptor
